# Association of high complement and low immunoglobulins with the clinical symptoms of patients with fibromyalgia

**DOI:** 10.1186/s13030-024-00321-9

**Published:** 2025-01-21

**Authors:** Satoshi Izuno, Masako Hosoi, Kozo Anno, Takahiro A. Kato, Nobuyuki Sudo, Kazufumi Yoshihara

**Affiliations:** 1 Department of Psychosomatic Medicine, Graduate School of Medical Sciences, Kyushu University, 3-1-1 Maidashi, Higashiku, Fukuoka, 812-8582 Japan; 2 Department of Psychosomatic Medicine, Kyushu University Hospital, Fukuoka, Japan; 3 Multidisciplinary Pain Center, Kyushu University Hospital, Fukuoka, Japan; 4 Department of Neuropsychiatry, Graduate School of Medical Sciences, Kyushu University, Fukuoka, Japan; 5 Center for Health Sciences and Counseling, Kyushu University, Fukuoka, Japan

**Keywords:** Fibromyalgia, Complement, CH50, Immunoglobulin, Fatigue, Anxiety

## Abstract

**Background:**

Fibromyalgia (FM) is a disease characterized by chronic widespread pain concomitant with various symptoms such as fatigue and anxiety. Although chronic inflammation has been implicated in the immunological abnormalities of FM, there are few human studies on complement and immunoglobulins. In this study, we investigated the immunological characteristics of FM patients and the association between their clinical symptoms and immunological indices, including complement and immunoglobulins.

**Methods:**

1) The serum 50% hemolytic complement activity (CH50), C3, C4, IgG, IgM, and hsCRP of 36 FM patients and 30 healthy sex- and age-matched controls (HC) were measured and compared. 2) Data from the Brief Pain Inventory (pain intention and pain interference subscales), the visual analog scale (VAS) (low back pain, knee pain, and fatigue), the State-Trait Anxiety Inventory and the Center for Epidemiologic Studies Depression Scale (anxiety and depression), and CH50, IgG, and hsCRP as immunological indices were acquired for 41 FM patients. Correlation analysis was done of the clinical symptom and immunological indices.

**Results:**

1) The FM group had significantly higher CH50 and lower IgG and IgM than the HC group after adjusting for body mass index (BMI). 2) Correlation analysis of immunological indices and clinical symptoms showed a positive, partial correlation between CH50 and fatigue and trait anxiety after adjusting for sex, age, and BMI.

**Conclusions:**

FM patients had higher CH50 and lower immunoglobulin levels than HCs. CH50 was also associated with the fatigue and trait anxiety of FM patients. Further studies are needed to determine whether changes in these immunological indices can be used as biomarkers and/or therapeutic targets for FM.

## Introduction

Fibromyalgia (FM) is a disease characterized by chronic widespread pain concomitant with various physical and psychiatric symptoms, such as fatigue, depression, and anxiety. It has been reported that the prevalence of FM in the general population is 2–3% worldwide and that it is common among middle-aged women [1]. FM significantly impacts quality of life and social activities and is associated with social problems such as work productivity loss and increased health care costs [2]. Data on immunologic, genetic, and psychosocial factors have accumulated that contribute to the etiology of FM, but there is currently no consistent pathogenetic model for this disease.

Recent reports on immunological factors associated with FM suggest that chronic inflammation caused by immune cells and inflammatory cytokines in the brain, spinal cord, and peripheral nerves leads to abnormal pain processing [3]. The complement system is composed of important proteins involved in defense against infection, and the association of high levels of complement with chronic inflammatory diseases such as rheumatoid arthritis and vasculitis syndrome has long been recognized. In recent years, complement abnormalities have been reported in various diseases, including neurological and psychiatric disorders [4, 5], and complement has been implicated in inflammation of the central and peripheral nervous systems. Animal studies have shown that complement is associated with symptoms of FM, such as pain and fatigue. It has been reported that several complements, including C3 and C4, are upregulated in rat models of neuropathic pain and that decreasing C3 suppresses pain behavior [6]. A study in mice found that intracerebroventricular administration of C3a inhibited morphine-induced analgesia [7], suggesting that C3a inhibits endogenous analgesia. In human studies, cluster analysis studies of blood data in chronic fatigue syndrome (CFS), a disease analogous to FM, found elevated C3 in clusters of severe and moderate cases compared to clusters of mild cases [8]. It has also been reported that CFS patients have elevated C4a and exacerbated fatigue with exercise [9].

Evidence is accumulating that suggests a link between complement and psychiatric symptoms. Elevated complement has been reported in patients with psychiatric disorders associated with depression and anxiety, such as major depressive disorder [10] and post-traumatic stress disorder [11]. An animal study reported that mice overexpressing C4A, one of the two isoforms of C4, have increased synaptic pruning by microglia, which results in decreased synaptic density and increased anxiety-like behavior [12].

In addition to complement, immunoglobulins have been implicated in the pathogenesis of FM. It has been reported that a high percentage of FM patients have low immunoglobulin levels [13] and that FM is more common in patients with primary immunodeficiency [14]. A study using proteomic analysis reported a significant decrease in IgG-related proteins in FM patients [15]. Regarding the association with pain, it is clear that the lower the IgG1 is in FM patients, the greater the degree of peripheral neuropathy [13].

Thus, the role of chronic inflammation in FM is becoming clearer, but few studies have compared the complement and immunoglobulins of FM patients with those of healthy controls (HC). We hypothesized that FM patients would have higher complement and lower immunoglobulin levels than HCs and that the higher the complement levels of FM patients the more pain, fatigue, depression, and anxiety they would experience and that the lower the IgG level the more pain they would experience.

Because high-sensitivity C-reactive protein (hsCRP) is widely used as an inflammatory marker and it has been reported that FM patients have higher hsCRP than HCs [16], we also performed an additional analysis of hsCRP.

## Methods

### Participants

The data of 41 Japanese FM patients who visited Kyushu University Hospital between 2014 and 2019 was available for study. All had peripheral blood immunologic findings taken and psychological indices administered during this period. The diagnosis of FM was made by an experienced psychosomatic physician following the American College of Rheumatology (ACR) 2010 diagnostic criteria [17]. According to these criteria, fibromyalgia is diagnosed when the following three criteria are met: 1) widespread pain index (WPI) ≧ 7 and symptom severity (SS) ≧ 5, including fatigue, waking unrefreshed, and cognitive symptoms, or WPI 3–6 and SS ≧ 9; 2) persistence of clinical symptoms for at least three months, and 3) absence of other conditions that would explain the chronic pain. HCs were 30 Japanese with no history of chronic pain or psychiatric disorders recruited from the local population by advertisement. All participants gave written informed consent before the study. The study was approved by the ethics committee of Kyushu University Hospital (26–406, 26–305).

### Measurement of immunological findings

50% hemolytic complement activity (CH50) was measured by liposome immunoassay; C3, C4, IgG, IgM by immunoturbidimetric assay; and hsCRP by nephelometry. All CH50 values above 60 U/mL were expressed as 60 U/mL due to limitations of the measurement system. Antinuclear antibodies (ANA) were measured using the fluorescent antibody method. Titers at or above 1/80 were considered positive.

### Assessment of clinical symptoms and psychological indices

Clinical symptoms were assessed for pain and fatigue characteristic of FM. Overall pain intensity was measured using the Brief Pain Inventory (BPI) [18, 19]. The BPI assesses two factors: pain intensity and interference (the degree to which pain interferes with activity, mood, sleep, etc.). Low back pain and knee pain were assessed as site-specific pain using a visual analog scale (VAS) of 0–100. Fatigue was also assessed using a VAS of 0–100. The severity of FM was assessed using the Fibromyalgia Impact Questionnaire (FIQ) [20, 21]. The FIQ focuses on various aspects of the condition, including physical functioning, work status, depression, anxiety, sleep, pain, stiffness, fatigue, and well-being. It is widely used internationally as a tool for assessing the overall impact of fibromyalgia on patients’ lives.

Psychological indices were given as self-administered questionnaires. The Japanese version of the Center for Epidemiologic Studies Depression Scale (CES-D) was used to assess depression [22, 23] and the Japanese version of the State-Trait Anxiety Inventory (STAI) to assess anxiety [24, 25]. The STAI assesses two types of anxiety: state anxiety (anxiety currently felt) and trait anxiety (tendency to feel anxiety).

The reliability and validity of the VAS (pain [26], fatigue [27]) and the Japanese versions of BPI [18, 19], FIQ [20, 21], and the psychological indices used (CES-D [22, 23], STAI [24, 25]) have been verified and reported.

### Statistical analysis

The data of 36 FM patients and 30 sex- and age-matched HCs were available for between group comparisons of demographic and clinical data and for the immunological findings. Normality was assessed by the Shapiro–Wilk normality test. Comparisons between the FM and HC groups were performed using Student’s t-test for normal distribution, Kruskal–Wallis test for non-normal distribution, and Pearson’s chi-squared test for sex and ANA positivity. To adjust for the effect of body mass index (BMI), a between group comparison of the immunological findings was performed using BMI as a covariate for FM patients and HCs, all of whom had height and weight measured within three months before and after the blood testing. The data of 31 FM patients and 30 HCs were available for analysis because height and weight data were missing for five of the FM patients. CH50 was missing for one of the FM patients, thus the comparison of CH50 was limited to 30 FM patients and 30 HCs. The comparison of hsCRP was limited to 26 FM patients and 30 HCs because the data for hsCRP was missing for five of the 31 FM patients. The other immunological findings of 31 FM patients and 30 HCs, corrected for BMI, were available for analysis.

The data of the full group of 41 FM patients, including the above 36, were available for correlation analysis of the immunological findings and clinical symptoms. Correlation analysis was performed using Spearman’s rank correlation test. For the items for which correlations were found between immunological findings and clinical symptoms, a partial correlation analysis was performed with Spearman’s rank correlation test using sex, age, and BMI as control variables for FM patients, whose height and weight were measured within three months before and after blood testing. Height and weight data were missing for six of the FM patients, leaving the data of 35 available for analysis. CH50 was missing for two patients, IgG was missing for one, and hsCRP was missing for seven of the 35 FM patients, which allowed partial correlation analysis of the CH50 of 33 FM patients, the IgG of 34 FM patients, and the hsCRP of 28 FM patients when corrected for BMI.

Partial correlation analysis was performed using MATLAB R2020a (Mathworks Inc., Natick, MA, USA), and other statistical analyses were performed using JMP Pro, Version 15 Software, SAS Institute Inc., Carolina, USA, with a statistical significance level of *p* < 0.05. One-tailed tests were used to test one-tailed hypotheses, and two-tailed tests were used for all others.

Three additional analyses were done: one excluding the one patient with SAS, another excluding the two patients with hypertension, and another excluding the one patient with SAS and the two patients with hypertension.

## Results

### Between-group differences in demographic and clinical data

Differences in the demographic data, clinical symptoms, and psychological indices of the 41 FM patients, 30 HCs, and 36 sex- and age-matched FM patients are shown in Table 1**.** Pain, fatigue, CES-D, and STAI scores were significantly higher in the FM group (each *p* < 0.001). There was no significant difference in sex, age, or BMI.

**Table 1:** Demographic and clinical characteristics

	HC (*n* = 30) (mean ± SEM)	Sex- and age-matched FM (*n* = 36) (mean ± SEM)	Total FM (*n* = 41) (mean ± SEM)	*p* value*
Sex (female, %)	90	89	78	0.88
Age (years)	42.3 ± 1.2	42.7 ± 1.8	43.2 ± 1.8	0.85
BMI (kg/m^2^)	21.9 ± 0.6	23.6 ± 0.8 ^1)^	23.5 ± 0.8 ^2)^	0.18
BPI intensity	1.2 ± 0.2	6.0 ± 0.2	6.0 ± 0.2	< 0.001
BPI interference	0.4 ± 0.1	6.2 ± 0.3	6.0 ± 0.3	< 0.001
Low back pain	13.1 ± 4.0	63.5 ± 5.6	62.8 ± 5.3	< 0.001
Knee pain	7.5 ± 3.1	53.3 ± 5.5	51.1 ± 5.4	< 0.001
Fatigue	32.3 ± 5.3	73.1 ± 4.0	73.3 ± 3.6	< 0.001
CES-D	5.2 ± 1.5	25.7 ± 1.4	24.9 ± 1.4	< 0.001
STAI (state)	34.8 ± 1.9	53.6 ± 1.9	52.7 ± 1.8	< 0.001
STAI (trait)	36.1 ± 1.6	56.9 ± 1.8	55.7 ± 1.7 ^3)^	< 0.001

1) *n* = 31, 2) *n* = 35, 3) *n* = 40

^*^ *p* values for comparison of HC and FM(*n* = 36) calculated with the Student t-test, Kruskal–Wallis test, or Pearson’s chi-squared test (Sex)

The duration of FM was 10.4 ± 1.5 years (mean ± SEM). Of the 41 FM patients, 80.5% were taking antidepressants, 78.0% were taking analgesics (56.2% pregabalin/gabapentin, 46.9% non-steroidal anti-inflammatory drugs, 31.3% acetaminophen, 28.1% opioids, and 28.1% neurotropin), and 17.1% were taking steroids (oral, inhalers, and topical medications). Of the 41 FM patients, 26 had physical comorbidities (6 obesity, 6 asthma, 4 dyslipidemia, 3 reflux esophagitis, 2 hypertension, 2 chronic thyroiditis, 2 atopic dermatitis, 1 sleep apnea syndrome (SAS), and others). The FIQ of 40 FM patients was 69.5 ± 2.5 (mean ± SEM), indicating severe FM (the data was missing for one of the patients).

### Between-group differences in complement and immunoglobulins

**Fig. 1:**
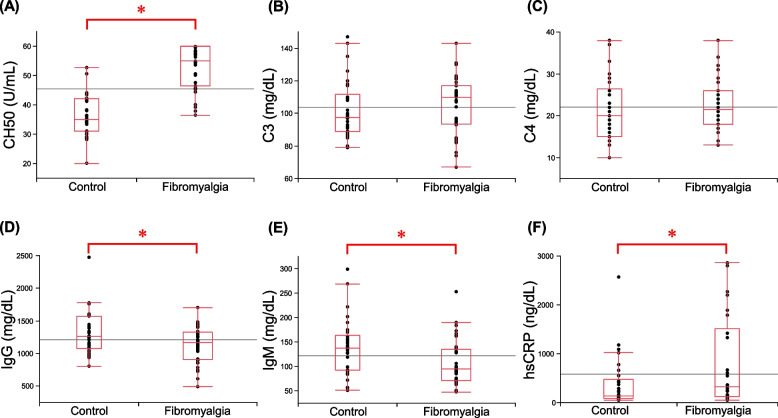
Immunological findings from patients with fibromyalgia and healthy controls. **A** Box-and-whisker plot showing the CH50 of FM patients (*n* = 35; 25th percentile, 46.5; median, 54.9; 75th percentile, 60.0) and healthy controls (*n* = 30; 25th percentile, 31.0; median, 35.0; 75th percentile, 42.1). **B** C3 of FM patients (*n* = 36; 25th percentile, 93.3; median, 110; 75th percentile, 117) and healthy controls (*n* = 30; 25th percentile, 88.8; median, 97.5; 75th percentile, 112). **C** C4 of FM patients (*n* = 36; 25th percentile, 18.0; median, 21.5; 75th percentile, 26.0) and healthy controls (*n* = 30; 25th percentile, 15.0; median, 20.0; 75th percentile, 26.5). **D** IgG of FM patients (*n* = 36; 25th percentile, 903; median, 1165; 75th percentile, 1327) and healthy controls (*n* = 30; 25th percentile, 1077; median, 1259; 75th percentile, 1575). **E** IgM of FM patients (*n* = 36; 25th percentile, 71.8; median, 95.0; 75th percentile, 136) and healthy controls (*n* = 30; 25th percentile, 92; median, 137; 75th percentile, 163). **F** hsCRP of FM patients (*n* = 26; 25th percentile, 130; median, 327; 75th percentile, 1512) and healthy controls (*n* = 30; 25th percentile, 86; median, 134; 75th percentile, 468). *p < 0.05

The immunological findings of the 41 FM patients, 30 HCs, and 36 sex- and age-matched FM patients are shown in Table 2, and individual results are shown in Fig. 1. CH50 was significantly higher in the FM group than in the HC group (*p* < 0.001) (Fig. 1A). In contrast, there was no significant difference in C3 or C4 (*p* = 0.16 and 0.29, respectively) (Fig. 1B,C). IgG and IgM were significantly lower in the FM group (*p* = 0.016 and 0.011, respectively) (Fig. 1D,E). The additional analysis showed that hsCRP was significantly higher in the FM group (*p* = 0.020) (Fig. 1F). There was no significant difference in ANA positivity.

**Table 2:** Immunological findings

	HC (*n* = 30) (mean ± SEM)	Sex- and age-matched FM (*n* = 36) (mean ± SEM)	Total FM (*n* = 41) (mean ± SEM)	*p* value*
CH50 (U/mL)	36.7 ± 1.4	52.9 ± 1.2 ^1)^	52.6 ± 1.1 ^2)^	< 0.001
C3 (mg/dL)	102.1 ± 3.3	105.2 ± 3.0	104.0 ± 2.8	0.32
C4 (mg/dL)	21.6 ± 1.4	22.5 ± 1.0	22.3 ± 1.0	0.59
IgG (mg/dL)	1316.7 ± 61.2	1120.9 ± 44.7	1119.5 ± 41.5 ^3)^	0.033
IgM (mg/dL)	139.6 ± 10.6	107.4 ± 7.8	103.6 ± 7.3 ^3)^	0.022
hsCRP (ng/dL)	381.4 ± 96.3	813.4 ± 180.4 ^4)^	853.3 ± 193.8 ^5)^	0.020
ANA positivity (%)	3.3	8.3	7.3	0.40

1) *n* = 35, 2) *n* = 39, 3) *n* = 40, 4) *n* = 26, 5) *n* = 30

^*^ *p* values for comparison of HC and FM (*n* = 36) calculated with the Student t-test, Kruskal–Wallis test, or Pearson’s chi-squared test (ANA positivity)

In the analysis with BMI as a covariate, CH50 was significantly higher (*p* < 0.001) and IgG and IgM significantly lower (*p* = 0.018 and 0.029, respectively) in the FM group than in the HC group. In contrast, there was no significant difference in hsCRP (*p* = 0.12).

The same result was obtained even after excluding two patients with hypertension (CH50 (*p* < 0.001), IgG (*p* = 0.024), IgM (*p* = 0.022), and hsCRP (*p* = 0.083)). One patient with SAS was not included in the 36 FM patients used for the between group comparison.

### Correlation of complement and immunoglobulins with the symptoms and psychological indices of patients with fibromyalgia

**Fig. 2:**
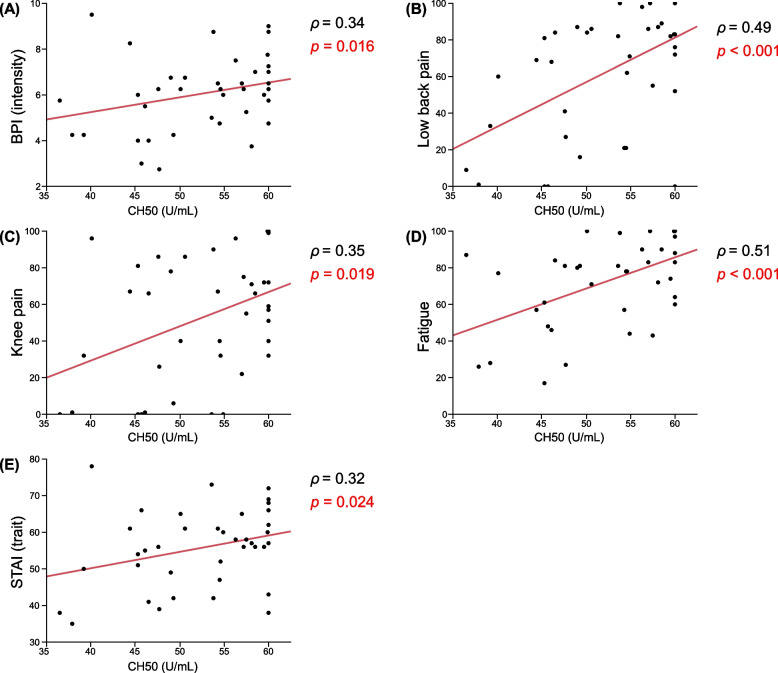
Correlation analysis of CH-50 and clinical scores. Correlation between CH50 of FM patients (*n* = 40) and several clinical scores (**A**) BPI (pain intensity); (**B**) Low back pain (VAS); (**C**) Knee pain (VAS); (**D**) Fatigue (VAS); (**E**) STAI (trait anxiety). *r* indicates the correlation coefficient

Positive correlations were found between CH50 and BPI (pain intensity) (*ρ* = 0.34, *p* = 0.016) (Fig. 2A), low back pain (*ρ* = 0.49, *p* = 0.001) (Fig. 2B), knee pain (*ρ* = 0.35, *p* = 0.019) (Fig. 2C), fatigue (*ρ* = 0.51, *p* < 0.001) (Fig. 2D) and trait anxiety (*ρ* = 0.32, *p* = 0.024) (Fig. 2E). Even after adjusting for sex, age, and BMI, the partial correlations were significant between CH50 and fatigue (*ρ* = 0.39, *p* = 0.016) and trait anxiety (*ρ* = 0.52, *p* = 0.002).

Positive correlations were found between hsCRP and low back pain (*ρ* = 0.48, *p* = 0.005) and knee pain (*ρ* = 0.44, *p* = 0.009), however, no significant partial correlation was found after adjusting for sex, age, and BMI.

In contrast, IgG had no significant correlation with the clinical symptoms or psychological indices.

The same result was obtained even after excluding one patient with SAS (CH50 and fatigue (*ρ* = 0.39, *p* = 0.019) and anxiety (*ρ* = 0.52, *p* = 0.002)), excluding two patients with hypertension (CH50 and fatigue (*ρ* = 0.40, *p* = 0.016) and anxiety (*ρ* = 0.60, *p* < 0.001)), and excluding one patient with SAS and two patients with hypertension (CH50 and fatigue (*ρ* = 0.40, *p* = 0.018) and anxiety (*ρ* = 0.61, *p* < 0.001)).

## Discussion

CH50, C3, C4, IgG, and IgM of 36 FM patients and 30 sex- and age-matched HCs were compared to examine possible correlations between their clinical symptoms and those of 41 FM patients. The results showed for the first time that FM patients had significantly higher CH50 and lower IgG and IgM than HCs, even after adjusting for BMI. In addition, we found for the first time that CH50 was significantly and partially, positively correlated with the fatigue and trait anxiety of FM patients, even after adjusting for sex, age, and BMI.

CH50 represents the total complement activation capacity due to reactions involving all complement components from C1 to C9. In general, CH50 can be elevated when production is stimulated by complement consumption due to chronic inflammation, such as that seen in some autoimmune diseases, infections, and malignancies. This study suggests that chronic inflammation is also involved in the pathogenesis of FM: CH50 elevation was higher in FM patients than in HCs. However, because CH50 reflects the total complement activation capacity, it will be necessary to identify which complement component is involved.

A proteomic analysis study, which provides a comprehensive analysis of protein structure and function, reported that C4A, a C4 isoform, was elevated in both the cerebrospinal fluid [28] and serum [15] of FM patients. In our study, no difference in C4 was observed between FM patients and HCs: C4a, a degradation product of C4, will need to be further examined because it is possible that even if C4 production is increased in FM patients C4 consumption may also be increased, resulting in a normal range.

This study also found significantly lower IgG and IgM in FM patients than in HCs, even after adjusting for BMI. A previous study reported that a higher percentage of FM patients had IgG and IgM levels below the lower limit of normal [13]. In this study, 8 of 40 (20%) FM patients had IgG below the lower limit of normal and 2 of 40 (5%) had IgM below the lower limit of normal (IgG data were missing for one of the FM patients, leaving the data of 40 available for this analysis.): 1 of 30 (3%) HCs had IgG below the lower limit of normal and none had IgM below the lower limit of normal, consistent with previous study. In addition, new findings show that IgG and IgM are significantly lower in the normal range compared to HCs.

There was no significant difference in ANA positivity between the FM and HC groups. Our findings are consistent with those of a previous study [29] that reported that the ANA positivity of FM patients and HCs was similar. It also reported that the frequencies of symptoms were not significantly different for ANA-positive and ANA-negative FM patients. MMP-3 is a proteinase produced by synovial cells and chondrocytes in the joint and is used as a biomarker for joint destruction in patients with rheumatoid arthritis. MMP-3 was not measured in this study. However, previous studies have reported that MMP-3 levels were normal in FM [30] and significantly lower than in rheumatoid arthritis and spondyloarthritis [31], suggesting that it is unlikely to be a useful biomarker for the symptoms in FM.

Partial correlation analysis of immunological indices and clinical symptoms showed that CH50 was significantly, positively correlated with fatigue and trait anxiety, even after adjusting for sex, age, and BMI. One study found elevated C4a and worsening fatigue in CFS patients after exercise [9]. In addition, treatment with complement-targeted drugs has been shown to improve the fatigue of patients with paroxysmal nocturnal hemoglobinuria [32] or cold agglutinin disease [33], suggesting a link between activation of the complement system and fatigue. In addition, because complement activation reduces the integrity of the blood–brain barrier, the possibility that interactions between complement and immune cells in the brain, such as microglia, contribute to neuroinflammation has attracted attention [34]. A study that used positron emission tomography (PET) with a radioligand that binds to the 18-kDa translocator protein to measure the microglial activity of FM patients reported that the more their fatigue, the more microglial activity was seen in the middle cingulate cortex [35].

Regarding the relation between complement and anxiety, a study examining the association between premenstrual syndrome symptoms and biomarkers reported a positive correlation between C4 and anxiety scores [36]. It is also known that microglia depend on complement for synaptic pruning [37], and it has been reported that mice overexpressing C4A have increased synaptic pruning by microglia, which resulted in decreased synaptic density and increased anxiety-like behavior [12]. Based on these studies and the present results, neuroinflammation due to microglial abnormalities as well as complement may be relevant in FM. Future studies are needed to examine the relations between complement and fatigue and anxiety, including the effects of neuroinflammation caused by microglial activation.

In this study, there was no correlation between CH50 and pain after adjusting for age and BMI. Complement components have been reported to be elevated in patients with pain, such as osteoarthritis and neuropathic pain, including C3a, C5, and C5a [38]. Our study examined the correlation between CH50, which reflects total complement activation capacity, and pain, thus methodological limitations may be the reason no association was observed.

We also found no correlation between IgG and pain. A decrease in intraepidermal nerve fiber density (IENFD), which is seen in small fiber sensory neuropathy involving Aδ- and C-fibers among peripheral nerves, has been reported in FM [39]. IENFD was reported to be lower in FM patients with lower IgG1 in a previous study [13]. An animal study reported that the administration of IgG from FM patients (FMIgG) to mice increased Aδ- and C-fiber sensitivity to noxious mechanical and cold stimuli and decreased IENFD [40]. The FMIgG used in the study was collected from patients in the United Kingdom and Sweden, and when the subclasses of serum IgG were compared to those of HCs, only IgG1 was significantly lower in Swedish FM patients. In our study, we did not measure subclasses: only the correlation between total IgG and pain: which may be why no association was found. FMIgG has been shown to bind to peripheral nerve neurons, satellite glial cells, and dorsal root ganglia [40], suggesting that the IgG of FM patients may act on peripheral nociceptive afferents to promote denervation and hyperalgesia. However, because the pain of FM patients is also thought to be influenced by abnormalities in the central nervous system [41], the amount of IgG likely to act on peripheral nerves may not adequately explain the pain. Further studies are needed to determine the role of low IgG in the pathogenesis of FM.

We also found that hsCRP was higher in FM patients than in HCs and that there was a positive correlation with pain, however, this was not significant after adjusting for sex, age, and BMI. Obesity is thought to be a proinflammatory state associated with elevated levels of hsCRP [42]. In a meta-analysis examining the relation between FM and obesity [43], the prevalence of obesity in FM was 35.7%, and an association was found between obesity and a range of symptoms, including pain and fatigue. These reports and our findings suggest that the association between hsCRP and the pain of the FM patients in this study was due to the influence of BMI.

SAS [44] and hypertension [45] are also considered to be proinflammatory states. Because the FM group included one patient with SAS and two patients with hypertension, we excluded them, performed the same analysis, and got the same results, which indicates that the results of this study were not influenced by SAS or hypertension.

These and previous reports have shown that, among the immunological indices, CH50 is significantly higher while IgG and IgM are significantly lower in FM. In addition, CH50 was found to be significantly associated with clinical symptoms such as fatigue and anxiety, suggesting that complement and immunoglobulins, which can be easily measured and are not affected by other factors such as BMI, should be considered in the treatment of FM. In addition, these findings and reports suggest that the immune system interacts with both peripheral and central pain mechanisms in the formation of symptoms in FM. In the peripheral nervous system, immunoglobulins cause neuroinflammation and immune-mediated nerve damage, leading to nociplastic pain. In fact, IgG from FM patients injected into mice caused increased pain sensitivity and altered pain nerve fiber density [40]. In the central nervous system, the activation of microglia leads to central sensitization, increasing pain and hypersensitivity, as well as causing fatigue and anxiety. To prove this hypothesis, it will be important to investigate the degree of neuroinflammation in the brains of FM patients using PET ligands for proteins expressed by activated microglia and to extract microglia from brain tissue or cerebrospinal fluid to study the interaction between microglia and complement. Furthermore, preclinical and clinical trials to assess the effects of drugs that suppress complement and microglial activity will also be useful.

This study has several limitations. First, as this was a cross-sectional study, we are not able to confirm that the immunological features identified are responsible for the clinical symptoms of FM. Longitudinal studies before and after treatment intervention would be desirable. Second, the preponderance of women among the participants may limit generalizability. Third, it should be noted that the results presented do not take into account the potential effects of medications and comorbidities other than proinflammatory states, such as hypertension and SAS. Finally, the missing hsCRP data points may have an impact on the reliability of the resulting findings.

## Conclusions

We found higher CH50 and lower IgG and IgM in FM patients than in HCs. In addition, CH50 was shown to be associated with the fatigue and trait anxiety of FM patients. Further studies are needed to determine whether changes in these immunological indices can be used as biomarkers and/or therapeutic targets for FM.

## Data Availability

The data that support the findings of this study are available on request from the corresponding author KY. The data are not publicly available due to their containing information that could compromise the privacy of research participants.

## Abbreviations

ANA: Antinuclear antibodies
BMI: Body mass index
BPI: The Brief Pain Inventory
CES-D: The Center for Epidemiologic Studies Depression Scale
CFS: Chronic fatigue syndrome
CH50: 50% Hemolytic complement activity
FM: Fibromyalgia
HC: Healthy control
hsCRP: High-sensitivity C-reactive protein
SAS: Sleep apnea syndrome
PET: Positron emission tomography
STAI: The State-Trait Anxiety Inventory
VAS: Visual analog scale
